# Accelerating the simulation of annual bifacial illumination of real photovoltaic systems with ray tracing

**DOI:** 10.1016/j.isci.2021.103698

**Published:** 2021-12-25

**Authors:** Marco Ernst, Georgia E.J. Conechado, Charles-Alexis Asselineau

**Affiliations:** 1 The Australian National University, School of Engineering, Canberra, ACT 2600, Australia

**Keywords:** Solar terrestrial physics, Engineering

## Abstract

Accurate modeling of bifacial illumination is critical to improve the prediction of the energy yield of bifacial solar systems. Monte Carlo ray tracing is the most powerful tool to accomplish this task. In this work, we accelerate Monte Carlo ray tracing of large solar systems by nearly 90%. Our model achieves root-mean-square error values of 7.9% and 37.2% for the front and rear irradiance compared against single-axis tracking field reference data, respectively. The rear irradiance modeling error decreases to 18.9% if suspected snow periods are excluded. Crucially, our full system simulations show that surrounding ground surfaces affect the rear irradiance deep into the system. Therefore, unit system simulations cannot necessarily ignore the influence of the perimeter of large installations to accurately estimate annual yield. Large-scale simulations involving high-performance supercomputing were necessary to investigate these effects accurately, calibrate our simplified models, and validate our results against experimental measurements.

## Highlights

• Modeling bifacial illumination of full photovoltaic systems using ray tracing
• Acceleration strategies reduce heavy ray tracing requirements by nearly 90%
• Surrounding ground affects bifacial illumination deep into the system
• Simulated annual rear illumination estimation error below 6.5%

## Graphical abstract

**Figure:**
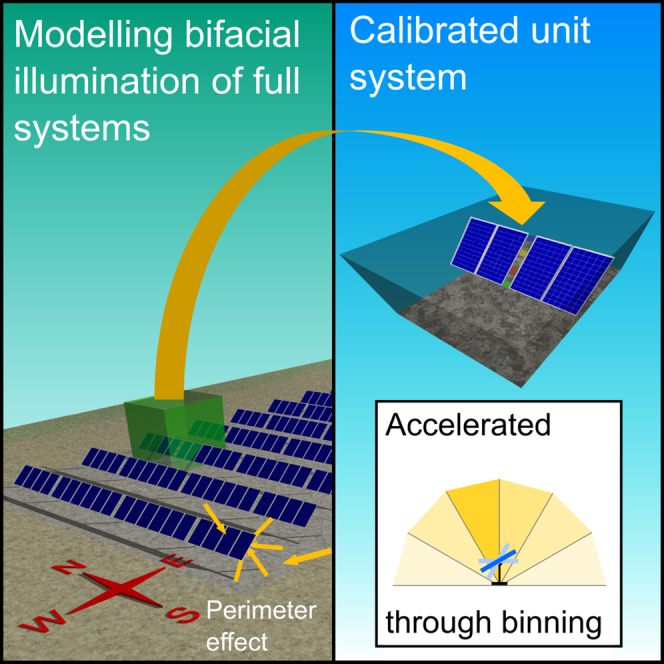

## Introduction

Bifacial solar photovoltaic modules can convert light into electricity from both sides and are gaining attention from solar farm developers because they offer higher energy yields without taking up additional space. Because of this technological advantage, the photovoltaic market is expected to be dominated by bifacial solar technology within less than a decade (ITRPV, 2021). The combination of bifacial solar modules with commercially available single-axis tracking systems can realize further increases in energy yield.

Although modeling the front irradiance on tilted planes is well understood (Sengupta et al., 2021; Hofmann and Sechmeyer, 2017; Ineichen, 2011), accurate modeling of rear irradiance is significantly more complex because of the interaction of light with many low-lying surfaces before it reaches the module. The level and uniformity of rear illumination depends on many factors including the direct-to-diffuse ratio, (spectral) ground albedo, ground-coverage ratio, array height, module location in the system, tracking algorithm, and structural system components such as mounting posts, mounting rails, and torque tubes in tracking systems. Crucially, many of these factors are time-dependent which complicates further the accurate prediction of rear illumination.

The expected yield gains from bifacial technology varies widely, ranging from 5% to 30% (Yusufoglu et al., 2014; Reise and Schmid, 2015; Pelaezet al., 2019a, 2019b), depending on the level and uniformity of rear illumination. Solar farm developers need accurate modeling of energy yields, especially from new technologies, to make accurate revenue and profit projections. Hence, accurate modeling of rear illumination is necessary to make informed decisions when developing large-scale solar systems.

Several studies have investigated a variety of optical effects in bifacial systems through modeling (Yusufoglu et al., 2014; Reise and Schmid, 2015; Pelaez et al., 2019a, Pelaez et al., 2019b, 2019c; Egido and Lorenzo, 1986; Pérez Oria and Sala, 1988; Lo et al., 2015; Lindsay et al., 2015; Shoukry et al., 2016; Chiodetti et al., 2016; Hansen et al., 2017; Janssen et al., 2017; Vogt et al., 2018; McIntosh et al., 2019; Horvath et al., 2019; Jäger et al., 2020). The two common methods for modeling the rear illumination of PV modules are view-factor (Yusufoglu et al., 2014; Egido and Lorenzo, 1986; Pérez Oria and Sala, 1988; Lindsay et al., 2015; Shoukry et al., 2016; Chiodetti et al., 2016; Hansen et al., 2017; Janssen et al., 2017; Jäger et al., 2020) and ray tracing (Reise and Schmid, 2015; Pelaez et al., 2019a, 2019b, 2019c; Lo et al., 2015; Vogt et al., 2018; McIntosh et al., 2019; Horvath et al., 2019). View-factor methods are used in established solar energy yield modeling software such as PVSyst (PVSyst, 2021); however, they tend to underestimate rear irradiance (Kang et al., 2019; Deline et al., 2019). They usually consider only a small number of optical effects, but typically disregard mounting components, and therefore require low computational resources. Edge effects and non-isotropic optical reflections are also usually not taken into account. Monte Carlo Ray Tracing (MCRT) on the other hand provides significant advantages for the modeling of complex optical properties or geometries, for example including system mounting components (Pelaez et al., 2019c; McIntosh et al., 2019) and are commonly used in the field of concentrated solar technologies for that reason (Wang et al., 2020). A few recent studies have presented ray-tracing results of PV modules. In Ref. (Pelaez et al., 2019c). Pelaez et al. utilize the ray tracing tool Radiance to model a full PV system (NREL, 2021b). Radiance is based on tracing rays of light backwards from a point of interest (e.g., sensor or solar cell location) to the sources (i.e., direct and diffuse irradiance component). McIntosh et al. performs forward ray tracing on unit PV systems (McIntosh et al., 2019). Backward ray tracing is typically more efficient than forward ray tracing when a few specific locations are of interest and becomes more computationally demanding for global illumination tasks. Irrespective of the ray-tracing method, limitations of computing requirements can be overcome by utilizing high-performance computing resources which are widely available through cloud-based services.

In this paper, we use an open-source forward MCRT method to simulate the bifacial irradiance of a single axis tracking system. We simulate and analyze rear illumination in full system simulations and use these results to calibrate a tailored unit system that accounts for the effects of torque tube, post shading and, crucially, the impact of the surrounding area. We validate our simulations against front and rear side irradiance measurements from a bifacial experimental single-axis tracking field managed by NREL (Pelaez et al., 2020). We further accelerate the MCRT simulations by applying a binning approach and a correction for the angle of incidence. Finally, we investigate the effects of measured time-resolved albedo values compared to an average albedo value and the effect of albedo anisotropy on MCRT simulations.

## Results and discussion

In this work, we perform MCRT simulations of front and rear illumination for full and unit PV systems. We first analyze the impact of the surrounding ground on the full system array. With this information, we then calibrate a unit system model using full system simulation results. The calibrated unit system is then used to analyze the impact of parameter binning strategy and resolution, and to critically evaluate the role of albedo properties on the simulation results and bias to measurements.

Fundamentally, MCRT is a stochastic simulation method in which complex interactions are approximated using randomly generated and propagated light rays. As any stochastic method, MCRT produces statistical outputs with inherent variability. Increasing the number of samples (i.e., the number of rays) decreases the range of this random uncertainty. In this study, the irradiance at each detector is subject to such random uncertainty. In this work, we report mean simulated irradiance values $\bar{I_{N}}$ and the corresponding random simulation error $CI\left(I_{N}\right)$ with a 95% confidence level as defined in the quantification and statistical analysis section.

### Effect of perimeter in full system MCRT simulations

Our MCRT model is built on the open-source Python-based software Tracer (Asselineau, 2021). Tracer enables a versatile implementation of the components of the PV system, for example solar modules with frames, tracker system torque tube and posts of arbitrary geometries. MCRT facilitates the simulation of complex radiant systems with many geometrical and optical properties. We assume isotropic reflection properties at module frames, mounting posts and torque tubes. The angular dependent specular reflections at the module glass are approximated with the Martin-Ruiz model (Martín and Ruiz, 2005). For the majority of our study we assume isotropic ground reflection; however, we demonstrate in Albedo specularity that ground reflectance anisotropy can affect the simulation results.

The basic geometry of the single-axis tracking system consists of mounting posts and torque tubes, solar modules, and the ground, illuminated by direct and diffuse sunlight. The geometric and optic system properties are listed in Table S1.

**Figure 1:**
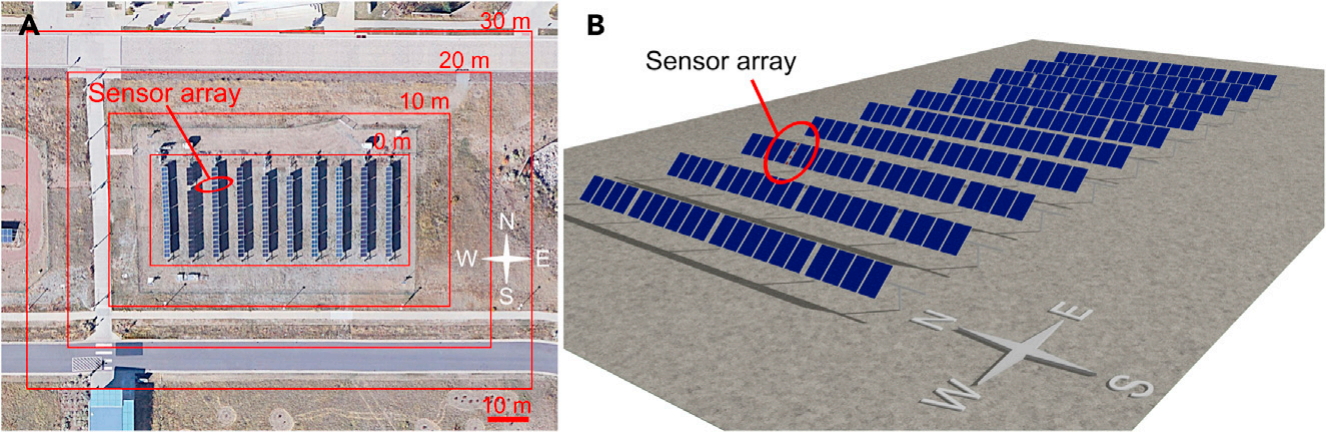
Satellite-image and 3D rendered scene of NREL photovoltaic systems modeled in Tracer (A) Satellite-image of NREL photovoltaic system indicating the position of the sensor array used in this work and varying perimeter size (Google Earth, 2019). (B) 3D rendered scene of full photovoltaic system modeled in Tracer.

Figure 1A shows the reference system located at NREL in Golden, CO, USA (Pelaez et al., 2020) indicating the position of front and rear irradiance sensors and the varying extent of considered ground area. This system is made up of both monofacial and bifacial modules and has several on-site plane-of-array irradiance sensors installed. The model validation is performed using the IMT (Ingenieurbüro Mencke & Tegtmeyer GmbH) silicon plane-of-array (POA) irradiance sensor array located in the third row from West four modules from North of the system. The modules in this row are monofacial, hence we assume isotropic reflection at the module backsheet. The sensor array consists of five sensors: one forward-facing sensor and four rear-facing sensors arranged along the length of the module.

Figure 1B illustrates the full PV system modeled in Tracer, consisting of ten identical module rows of each 20 modules, including posts, torque tube, and sensor array. The posts are located at each end of the rows and at modules 4, 10, and 16 from north. We model illumination from direct and isotropic diffuse sunlight, considering the sun position and module tracking angles.

Full system simulations are performed to determine the impact of the perimeter around the PV system and to serve as reference of subsequent calibration of unit system simulations. We vary the perimeter of the full system simulations for the six scenarios from 0 m to 30 m as illustrated in Figure 1A. The system with nominally 0 m perimeter includes the mounting tube and post that extend beyond the module area and half of a row-to-row spacing on the East and West sides of the system.

The full system is simulated in six tracking angle configurations, −50°, −30°, −10°, 10°, 30°, and 50° and corresponding sun position, with varying perimeter size, and for direct and diffuse sunlight assuming a constant ground albedo of 26% (cf. Albedo time resolution impact on module rear irradiance).

**Figure 2:**
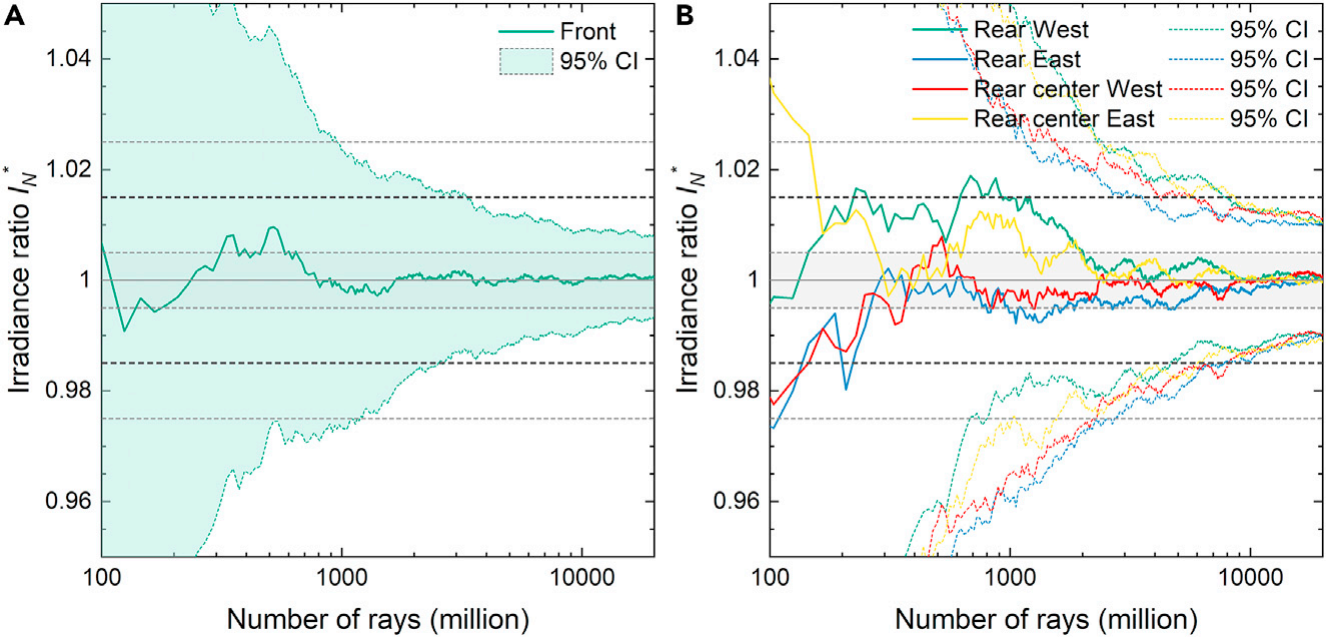
Front and rear mean relative difference in irradiance versus number of rays for the full system simulations (A and B) (A) Front and (B) rear mean relative difference in irradiance and corresponding confidence interval as function of number of rays for the full system simulations with 30 m perimeter.

Figure 2 shows (a) the front and (b) the rear irradiance results as a function of the number of rays cast for the 30 m perimeter case. In Figure 2, the mean ratio $I_{N}^{*}$ and corresponding error $\Delta I_{N}^{*}\Delta {\text{I}}_{\text{N}}^{\text{∗}}\Delta {\text{I}}_{\text{N}}^{\text{∗}}$ are the average results of the six configurations calculated separately. $I_{N}^{*}$ and $\Delta I_{N}^{*}\Delta {\text{I}}_{\text{N}}^{\text{∗}}\Delta {\text{I}}_{\text{N}}^{\text{∗}}$ are computed using Equations (5) and (6):

(Equation 1) $$I_{N}^{*}=\frac{\bar{I_{N,\text{ref}}}}{\bar{I_{N_{\text{max}},\text{ref}}}}$$

(Equation 2) $$\Delta I_{N}^{*}=\frac{\bar{\text{CI}\left(I_{N,\text{ref}}\right)}}{\bar{I_{N_{\text{max}},\text{ref}}}}$$

**Figure 3:**
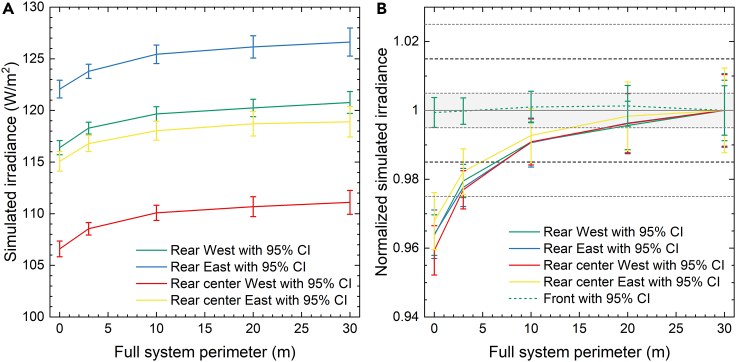
Absolute and normalized simulated irradiance versus the system perimeter (A and B) (A) Absolute and (B) normalized simulated irradiance as function of the system perimeter.

21,234 million rays were traced in each configuration and illumination type to obtain simulated irradiance uncertainty below 1.0%. This large number of rays is a direct consequence of the complexity of the simulation domain of the full reference system: a large scene extending over an area ranging from 1,700 m^2^ in the 0 m perimeter case to over 10,500 m^2^ with 30 m perimeter case and with over 680 optically interacting surfaces. It should be noted that rather than the actual size of the simulation domain, the number of geometrical elements simulated is what ultimately increases the computational load of ray-tracing simulations. However, the required number of rays scales linearly with the system footprint to maintain a constant ray density and thus constant uncertainty for a scene of similar complexity. With this in mind, more than 1 trillion rays were cast to obtain the data presented in Figures 2 and 3.

Figures 3A and 3B show the absolute and normalized irradiance detected at the sensor locations (average of the six tracking configurations) as a function of the perimeter. We can observe that above a perimeter width of 20 m the detected rear irradiance becomes relatively stable. The front irradiance (only shown in Figure 3B) is not significantly impacted by the perimeter size. The modeled rear irradiance sensors are located in the third row of modules, four modules from the north and 16 modules from the south. Despite this large distance from the system edges, the surrounding area still affects the results from a distance of more than 10 meters.

We note that all our simulations assume a spatially uniform albedo. In the real system, however, ground reflection is further affected by varying albedo and objects such as inverter stations and fences.

### Calibration of unit system MCRT simulations

Full PV plants can be subdivided into identical elementary unit systems that are repeated throughout the field. MCRT simulations of such PV unit-systems can reduce the simulation complexity and increase simulation speed but may lead to a systematic bias error because of a disproportionate impact of system components and/or perimeter of the system. In this work, we model the bifacial irradiance measured by the sensor array mounted on four modules from the system North edge. We define a PV system with four modules as our unit system and calibrate the sensor response to the full system simulations. We then determine the required number or rays per simulation to reduce the simulation error.

**Figure 4:**
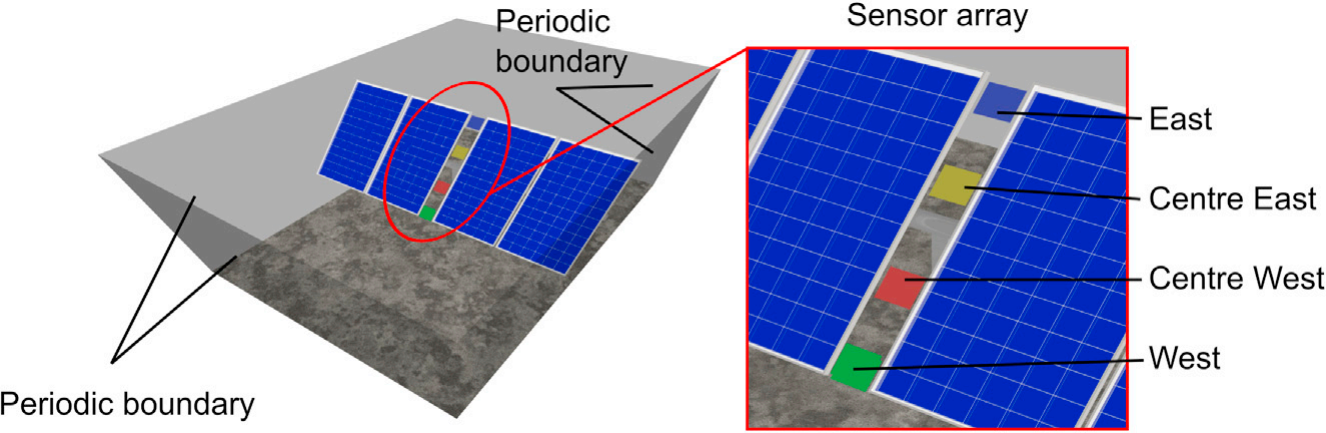
Illustration of 4-module photovoltaic unit system with periodic boundary conditions

Figure 4 shows the four-module unit PV system mounted on a torque tube and a central post replicating the full NREL system modeled in Tracer, included in an enclosure with periodic boundary conditions. The irradiance sensors are color-coded based on their position alongside the module: Green represents West, red represents center West, yellow represents center East, and blue represents East. In this example, the unit system approach results in modeling an infinitely large PV system with posts placed every four modules.

The measurement accuracy of IMT silicon irradiance sensors (IMT Solar, 2021) is stated with ±5 W/m^2^ and ±2.5% from the measured value. Hence, we aim to trace as many rays required to reduce the systematic and random error at the irradiance sensors to at least below 2.5%.

The calibration of the unit system is performed by simulating the same six configurations as previously, with tracking angles from −50° to 50° and constant ground albedo of 26%. We compare the unit system simulations against the full reference system simulations with a 30 m perimeter.

#### Optimization of unit system perimeter

**Figure 5:**
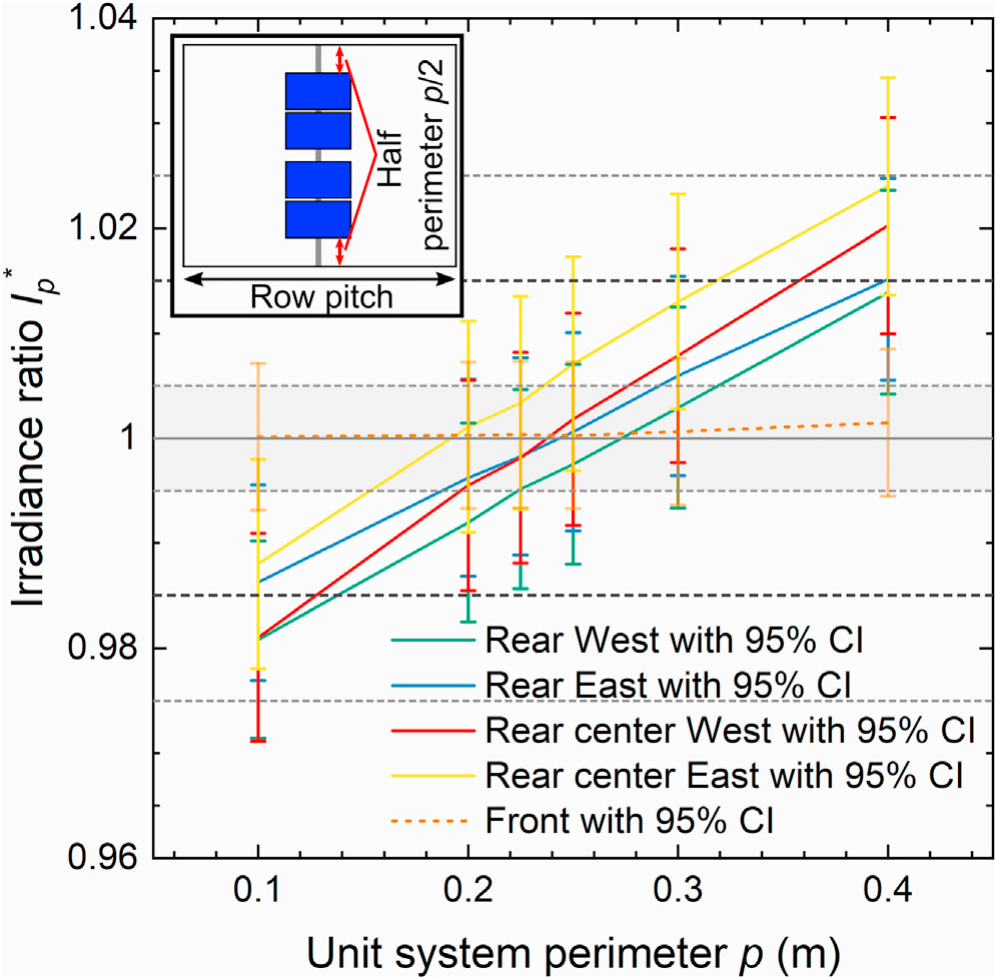
Front and rear irradiance ratio as function of the unit system perimeter

To take the impact of the surrounding perimeter of the full system into account in the unit system, additional ground areas are added to the North and South boundary of the unit system as illustrated in the inset of Figure 5. We note that this calibration is only valid for the specific location of the sensor array in the system. The impact of the perimeter would increase toward the edge of the system and decrease toward its center; however, without sensor data at such locations, it is impossible to validate simulation results.

Figure 5 shows the mean irradiance ratio simulated for the detector areas at (a) the front and (b) the rear as a function of the unit system perimeter. We determine the relative difference for each scenario from the difference of $\bar{I_{N_{\text{max}},p}}$ to the irradiance $\bar{I_{M_{\text{max}},\text{ref}}}$ for the full PV system reference simulation with 30 m perimeter. Here p refers to the perimeter of the unit system, and $N_{\text{max}}=1,000$ million and $M_{\text{max}}=21,234$ million to the maximum number of rays simulated in each configuration and illumination type for the unit and reference system, respectively.

(Equation 3) $$I_{p}^{*}=\frac{\bar{I_{N_{\text{max}},p}}}{\bar{I_{M_{\text{max}},\text{ref}}}}$$

Both – the unit system and the reference system – simulations are subject to statistical uncertainty. We thus calculate the resulting 95% confidence interval $CI\left(I_{p}^{*}\right)$ of $I_{p}^{*}$ by combining the individual uncertainties using Equation (8). The mean relative difference $\bar{I_{p}^{*}}$ and corresponding uncertainty $\bar{CI\left(I_{p}^{*}\right)}$ for each detector is then determined from the average of the six simulated scenarios.

From Figure 5 we can observe that the bias of all front and rear sensors is below 0.5% for a unit system perimeter of 0.225 m. Therefore, all following simulations will be performed using the unit system with this perimeter. Not surprisingly, the bias of the front sensor is practically independent of the perimeter size. The difference in bias of the four rear sensors stems from the distinct influence of posts, sensors, and irregular module gap which affects the periodic unit system differently than the full system.

#### Optimization of number of rays

**Figure 6:**
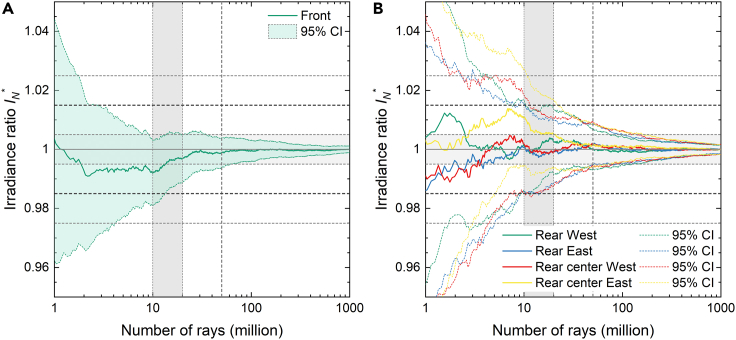
Front and rear mean relative difference in irradiance versus number of rays for the unit system simulations (A and B) (A) Front and (B) rear mean relative difference in irradiance and corresponding confidence interval as function of number of rays for the unit system simulations.

We now determine the required number of rays to reduce the statistical uncertainty below 1.5% in the unit system. Figure 6 shows the mean ratio $I_{N}^{*}$ and corresponding error $\Delta I_{N}^{*}$ for the average of the six scenarios calculated separately for the four detector areas at (a) the front and (b) the rear as a function of number of rays.

Based on Figure 6, we can conclude that approximately 10–20 million rays are sufficient to reduce the simulation error of the rear irradiance between 1.5% and 2.5% highlighted by the gray shaded area. This agrees with findings in Ref (McIntosh et al., 2019). for the number of rays in single-module unit systems. To account for possible scenarios with low ground albedo, all following simulations are performed with 50 million rays which reduces the expected error to less than 1.0%. This error calculation considers both direct and isotropic diffuse light. The number of rays could be further optimized independently for each irradiance contribution.

Comparison between Figures 6 and 2 highlights the advantage of unit system simulations compared to full system simulations. To reduce the random error of the full system simulation with 30 m perimeter to similar confidence values of 1.5%; we require nearly three orders of magnitude as many rays. Hence, using a well calibrated unit system is an effective tool for reducing the computational requirements.

### Influence of parameter binning resolution

Critically, MCRT computing time strongly depends on the number of rays simulated and scene complexity. In addition, numerous configurations are necessary to evaluate the annual performance of the system of interest. The number of configurations depends on several time-dependent parameters, such as sun position, tracking angle, and ground albedo.

In order to minimize the number of required configurations, we apply a parameter binning approach. First, direct and isotropic diffuse illumination are treated separately. Isotropic diffuse illumination does not depend on sun position; therefore, the number of binning parameters is reduced in this case. Input parameter ranges are split into a number of discrete parameter bins. The input parameters for a full annual simulation are then regrouped into the parameter bins that contain their specific value. Simulations are only performed for the mean value of each bin and results are obtained by interpolation from the bin results. In this work we use nearest-neighbor interpolation in the parameter space, for simplicity. Each direct and diffuse simulation is performed for a constant nominal direct normal irradiance (DNI) and diffuse horizontal irradiance (DHI) of 1000 W/m^2^, respectively.

The MCRT outputs for each simulation are the front-irradiance and rear irradiance at each sensor location illustrated in Figure 4. As a result of the binning approach, these values correspond to a series of sun position and tracking angle input that are mapped back to the full time-step dataset. The separate treatment of direct and diffuse illumination allows to independently reuse the simulations in a post-processing step. The simulation results at nominal 1000 W/m^2^ are weighted by the relevant irradiance at each time-step as described in Method details. The simulated sun position and tracking angles may differ from the annual dataset inputs, leading to a deviation between the simulated and actual angle of incidence at each timestep. To address this discrepancy, we apply an angle of incidence (AOI) correction factor *f* in Equation (4) to the direct irradiance simulation, taking into account the sun's incidence angle at the tracker plane relative to the sun's incidence angle of the corresponding simulated bin.

The publicly available single-axis tracking system field data installed at NREL in Golden, CO, USA (Pelaez et al., 2020), shown in Figures 1A and 1B were used to validate our models. Table S2 lists the time-resolved parameters from the dataset that are used in this study.

Because no DNI and DHI values are measured on site, we use the data provided by the NREL Solar Radiation Research Laboratory (SRRL) station which is located within 100 m from the PV system and included in the dataset. It is expected that this approximation introduces systematic errors, particularly in the case of partial cloud cover. Data filtering is applied to remove erroneous and/or unwanted entries, resulting in a dataset with 5326 valid timesteps.

We establish five bin groups with varying combinations of bin sizes listed in Table 1 along with the resulting number of required simulations for direct and isotropic irradiance, both with constant and time-resolved ground albedo assumptions. The bin sizes correspond to the resolution of the binning approach, with coarser resolution leading to larger bins and thus fewer simulations required. The relative acceleration achieved by the binning approach is calculated based on the required number of simulations compared to the full dataset. In the following results, we will refer to these five groups as “Full,” “Fine,” “Medium,” “Coarse,” and “Extra Coarse”.

**Table 1:** Simulation parameters for five bin groups

		Bin groups				
		Full	Fine	Medium	Coarse	Extra coarse
Bin sizes	Sun zenith/azimuth and tracking angle	1°	5°	10°	15°	20°
	Ground albedo	0.01	0.05	0.1	0.15	0.2
Number of simulations w/o measured albedo	Direct irradiance	2823	595	191	100	60
	Isotropic diffuse irradiance	120	24	12	8	6
Reduction of required number of simulations		44.7%	88.4%	96.2%	98.0%	98.8%
Number of simulations w/measured albedo	Direct irradiance	4126	1334	526	288	174
	Isotropic diffuse irradiance	1425	296	103	47	30
Reduction of required number of simulations		−4.2%	69.4%	88.2%	93.7%	96.2%

In Table 1 we observe that the binning approach can significantly reduce the number of required simulations. For example, at “Medium” resolution without albedo, we need to simulate a total of 203 configurations compared to represent the full dataset with 5326 timesteps – this corresponds to a 96.2% reduction of required number of simulations. We note that the separate treatment of direct and diffuse light can lead to an increase in the number of required simulations at small bin sizes, as in the case of the “Full” resolution when considering the time-resolved albedo.

The utilization of measured albedo values more than doubles the required number of direct irradiance simulations for each binning group and increases the number of isotropic diffuse simulations by a factor 3 to 6. Such an increase in number of simulations could translate to intractable computation times and we therefore perform a sensitivity analysis to quantify the impact of binning resolution and measured albedo on MCRT simulation accuracy.

In the following sections, three error metrics are used to compare the simulated and measured irradiance of the five sensors (one facing the sun, four facing the ground), the relative Root-Mean-Square Error (rRMSE), relative Mean Bias Error (rMBE), and Coefficient of Determination ($R^{2}$) as detailed in the quantification and statistical analysis section.

rRMSE measures accuracy as a percentage and is proportional to the size of the squared error; therefore, larger errors have a larger impact. rMBE provides the average error between the modeled and the measured value and is bias sensitive. $R^{2}$ similar to rRMSE is a measure of the squared error, but also assesses the quality of the model. For $R^{2}=1.0$, the simulation estimates and the observed values match perfectly, whereas negative values indicate an absence of correlation between the simulated and the measured data.

The error metrics for the front irradiance are compared against Hay and Davies' model (Hay and Davies, 1978) implemented in pvlib python (Holmgren et al., 2021; Holmgren et al., 2018). This comparison enables the evaluation of the effects of the spatial distance between the SRRL irradiance model inputs (from the nearby weather station) and the modeled POA irradiance at the system site.

We discuss the key results in the following sections of this paper. The complete error results are listed in Tables S3–S6.

**Figure 7:**
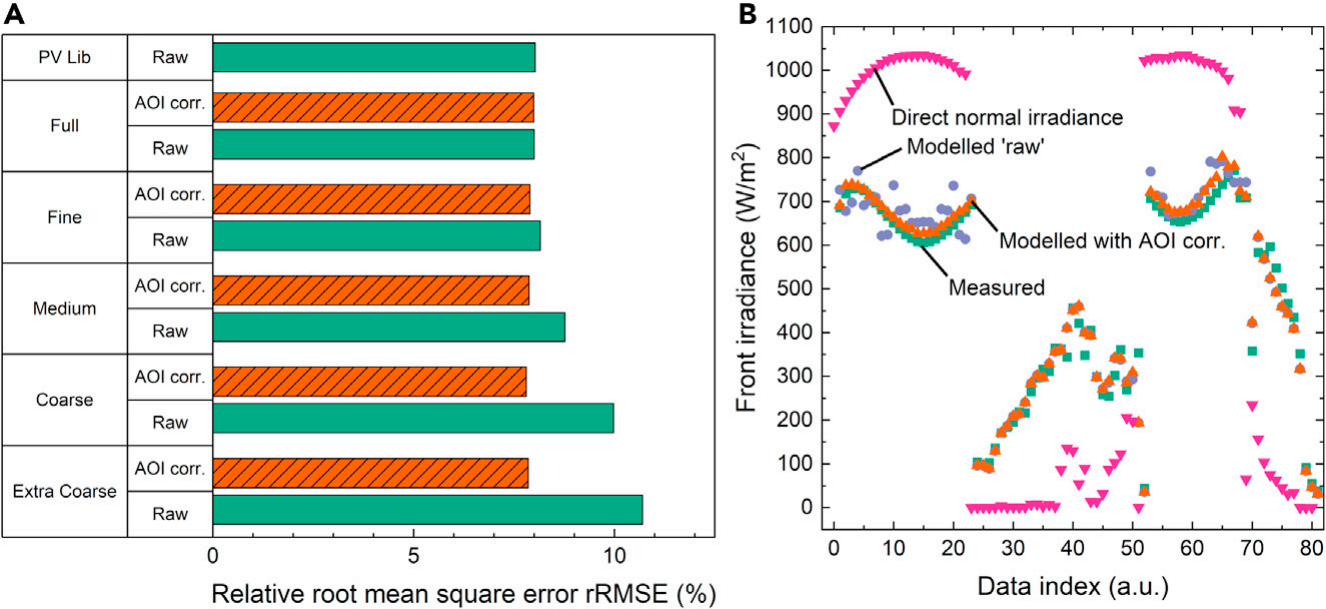
Impact of binning resolution and AOI correction on the front irradiance simulation error (A) Relative root-mean-square error for front irradiance simulations without (green bars) and with (hashed orange bars) AOI correction. Lower values are better. (B) Measured (green squares), modeled front irradiance with applying AOI correction (orange triangles) and without AOI correction (blue circles), and direct normal irradiance.

In Figure 7A we compare the rRMSE of the front irradiance as a function of the binning resolution. In particular, we show the impact of the AOI correction presented earlier. For the un-corrected “raw” simulations the error improves substantially with increased binning resolution. Only full resolution achieves similar rRMSE as the PV Lib reference irradiance simulations. In contrast, when applying the posterior AOI correction, the rRMSE is virtually independent of the binning resolution achieving consistent rRMSE values of 7.9%, thus enabling a significant reduction in simulation configurations. Consequently, the required binning resolution is determined only by the accuracy requirements of the rear illumination.

The effect of the AOI post-processing step on the modeled front irradiance is shown in Figure 7B for a partial dataset simulated at ‘Medium’ binning resolution. Application of the AOI correction leads to excellent match with experimental data for both high and low irradiance conditions.

**Figure 8:**
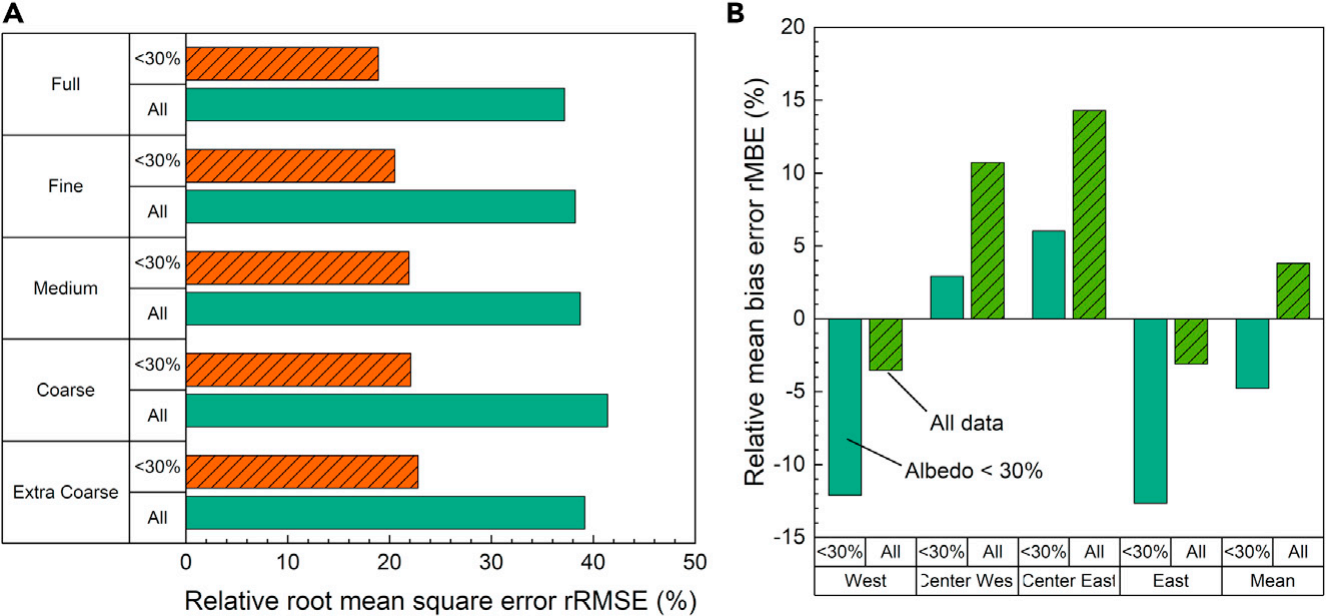
Impact of binning resolution and albedo threshold on the rear irradiance simulation error (A) rRMSE for rear irradiance simulations at different binning resolution for datapoints with albedo <30% (hashed orange bars) and full dataset (solid green bars). (B) rMBE (green bars) and rRMSE (orange and yellow bars) for each of the four rear-facing irradiance sensors for datapoints with albedo <30% (hashed bars) and full dataset (solid bars).

**Figure 9:**
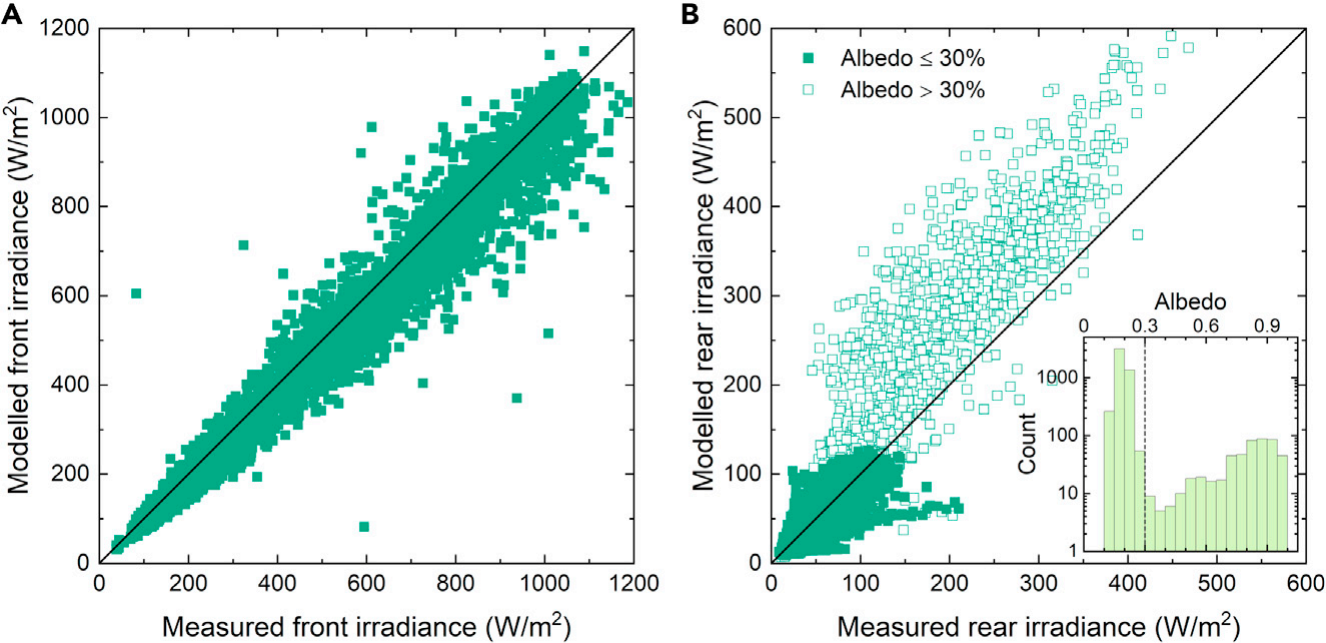
Modeled versus measured front and rear irradiance Modeled versus measured (A) front and (B) rear irradiance. Rear irradiance is shown for measured albedo values ≤30% (solid symbols) and albedo values >30% (open symbols).

Figure 8A shows the average rRMSE of the four rear sensors at the different binning resolutions. The AOI correction is not applied on rear sensors because they do not receive direct sunlight. Error metrics are computed for all 5326 data points and for a subset of data determined by excluding simulations with measured albedo higher than 30%. This 30% limit was chosen based on the albedo histogram in the inset of Figure 9B to filter out suspected periods of snow ground coverage. The filtering reduces the number of data points by ∼10%–4833; however, it has a significant impact on the rRMSE shown in Figure 8A. Interestingly, comparison with Figure 7A reveals that the binning resolution affects the rear irradiance to a lesser extent than the front irradiance. The best rRMSE for the entire dataset at ‘Full’ resolution is 37.2%, which increases to 41.4% at ‘Coarse’ resolution. The rRMSE appears to improve at the ‘Extra Coarse’ resolution, possibly because of the precise combination of the simulated angular configurations of the system.

Considering only albedo values ≤30% almost halves the rRMSE. An increase in binning resolution correlates with a decrease in rRMSE from 22.8% at ‘Extra Coarse’ resolution to 18.9% at ‘Full’ resolution.

The above error results are based on the mean values for the four rear facing sensors. However, Figure 8B reveals that there is considerable variation between the individual sensors results for the ‘Full’ binning resolution. The individual sensor simulation results either overestimate (positive rMBE) or underestimate (negative rMBE) the measured value. In these simulations, the East and West sensors, at the edge of the module, suffer stronger bias error than the central sensors.

Analysis of these error metrics highlight the complexity of the modeling undertaken: Direct and diffuse light may undergo reflections at several surfaces before being absorbed by a rear facing sensor. Unknown reflection properties at different surfaces and any non-uniformity of the scene, e.g., ground and surface reflection anisotropy, temporally varying albedo, illumination anisotropy, etc. are all compounded sources of error.

Despite a considerably higher number of required simulations, the ‘Fine’ binning resolution provides only marginal improvements over the ‘Medium’ binning resolution. Therefore, all subsequent simulations are performed at ‘Medium’ resolution.

Figure 9 shows the modeling results at ‘Medium’ binning resolution of front and rear irradiance versus the measured values. The distribution is wider at higher irradiance values, in particular evident for the rear illumination. The periods when rear irradiance exceeds approximately 150 W/m^2^ correlate with periods of albedo values above 30%. The 30% threshold was chosen based on the right edge of the first peak in the histogram, shown as an inset in Figure 9B. Further exploration of the relationship between albedo levels and simulation error is presented in the next section.

Using our simulation results, we determine a time-averaged ratio of rear irradiance to front irradiance received at the sensors of 11.7% and 9.1% for the full and <30% albedo limited datasets, respectively. This agrees well with the time-averaged ratios of 11.0% and 9.3% found for the measurements and results in 6.4% relative error for the full dataset and 2.2% when snow periods are excluded.

### Albedo time resolution impact on module rear irradiance

The previous section made use of the time-resolved albedo measurements available in the NREL dataset. However, such data is not usually available to solar farm developers and the MCRT model would typically need to rely on a constant “average” site albedo parameter. In this section we therefore investigate the impact of constant ground albedo on the rear illumination results from MCRT modeling. All error metrics calculated in this section are for the average of the four rear irradiance sensors.

The average POA irradiance-weighted albedo of the filtered dataset is 23.4%. Without POA irradiance weighting the average site albedo is 24.3%. In addition to local data, we explore how time-resolved satellite-based albedo data affects the quality of simulation results. For the satellite albedo values we use the surface albedo measurements from the MERRA-2 Radiation Diagnostics (M2T1NXRAD) dataset (Gelaro et al., 2017). This albedo data is available in a resolution of 0.5 ° × 0.625 ° in latitude and longitude, respectively, and hourly time resolution. We perform a spatial and temporal linear interpolation of the satellite-data to the location and 15-min timesteps of the NREL bifacial system.

**Figure 10:**
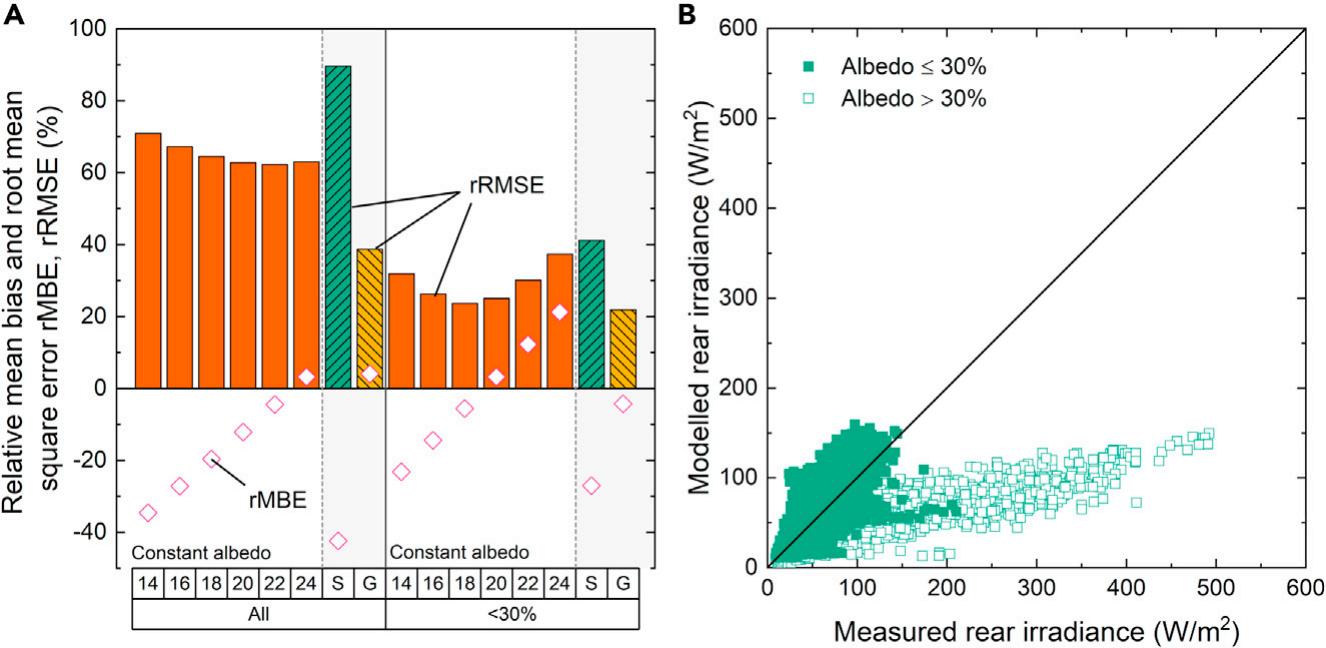
Impact of constant albedo parameter and albedo threshold on the rear irradiance simulation error (A) rMBE (open symbols) and rRMSE of rear illumination as function of constant albedo parameter (solid orange bars) compared to satellite- (S, hashed green bars) and ground-measured (G, hashed yellow bars) albedo results in gray-shaded regions. (B) Modeled versus measured rear irradiance for measured albedo values ≤30% (solid symbols) and albedo values >30% (open symbols).

Figure 10A shows the value of the error metrics versus albedo, with and without the <30% threshold and considers:

• Constant albedo. We vary the constant albedo to determine the optimum average albedo value.
• Time-resolved satellite-based albedo data (labeled ‘S’).
• Ground-measured time-resolved albedo (labeled ‘G’).

For the full dataset, the rMBE strongly depends on the average albedo value. The lowest rRMSE of 62.3% is achieved at an average albedo of 22%. When excluding datapoints with albedo greater than 30% the error metrics significantly improve and the difference with time-resolved simulation is reduced. The best rRMSE of 23.6% for an average albedo of 18% is close to the rRMSE of 21.9% for time-resolved albedo achieved at the same binning resolution.

In both cases, the satellite-albedo data perform significantly worse than local average albedo data. Despite the reduced rRMSE when applying the 30% albedo threshold, the low rMBE and negative R^2^ value (see Table S5) show that there is no correlation between the simulated and measured rear irradiance. This is also evident in Figure S1, where the satellite albedo doesn't correlate with the on-site albedo.

The higher rRMSE for the full dataset is mainly caused by the large deviation between the model and the measurement at times when the albedo is high: In Figure 10B we plot the modeled rear irradiance versus the measured rear irradiance for the best overall average albedo of 22%. We highlight the datapoints which have a measured albedo above the 30% with open symbols.

Fundamentally, the lack of accuracy of high albedo simulations has its source in the unknown optical properties of the ground in these conditions. Although spatially non-uniform coverage of snow and/or rain is a factor, the lack of data on the bidirectional reflection functions of the ground in these specific situations imposes simplifications on the model that lead to inaccuracies. Therefore, in the following section we further investigate the effect of non-isotropic ground reflections.

### Albedo specularity

In all previous simulations we assumed isotropic ground reflection. In this section, we apply specularity factor *f*_s_ to model an exemplary anisotropic ground reflection. *f*_s_ = 0% expresses a fully isotropic reflection as in the previous sections, *f*_s_ = 100% defines a fully specular reflection. We note that MCRT considers shading effects of the ground by modules, posts, and torque tube in all simulations.

**Figure 11:**
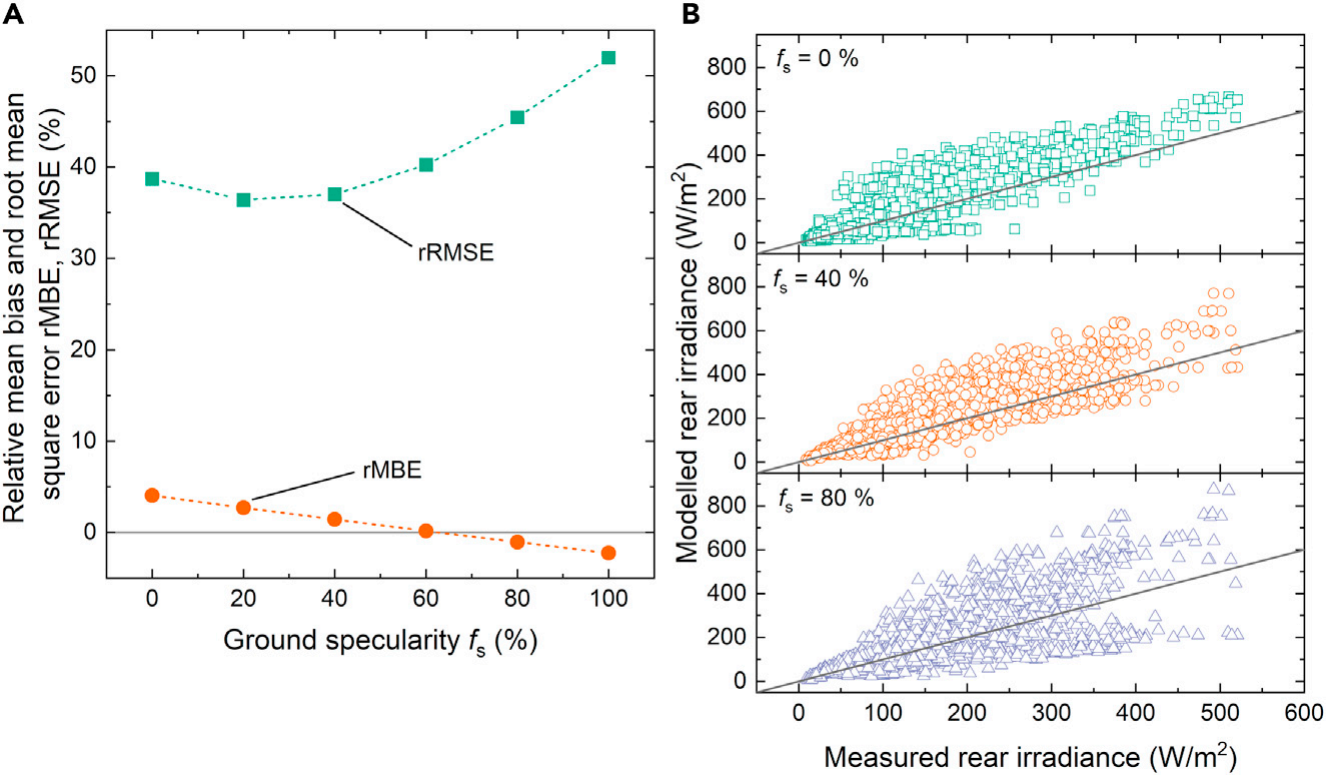
Impact of ground specularity on rear irradiance simulation error (A) rRMSE (green squares) and rMBE (red circles) of modeled rear irradiance as function of the ground specularity fs applied to albedo values >30%. (B) Modeled versus measured rear irradiance for albedo values >30% for fs = 0% (green squares), fs = 40% (red circles), fs = 80% (purple triangles).

In Figure 11A we plot the rRMSE and rMBE for the full time-resolved dataset simulated at ‘Medium’ binning resolution. Note that *f*_s_ is applied only to albedo values over 30%. The optimum specularity factor of 20% leads to a small improvement in the error metrics with an rRMSE as low as 36.4%.

Figures 11B illustrates the effects of specular reflection on the modeled rear irradiance. At *f*_s_ = 0%, the plot is identical to the data shown in Figure 9B. As specularity increases, we observe an increasing bimodal distribution of points. Although some points are shifted to higher values, others are shifted to lower values. This behavior can be explained by shadowing effects of specularly reflected light. Although the application of a specularity factor results in only a small improvement in the error metrics, the results demonstrate that albedo anisotropy can have a strong effect on the modeled rear irradiance.

### Conclusions

In this work, we applied an open-source MCRT package to model the bifacial irradiance of full and unit photovoltaic systems. Unit system simulations allowed us to significantly reduce the computational requirements while still providing accurate results for the whole system by calibration to full system simulations.

It is remarkable how important the influence of the surroundings is on the simulations of the rear side illumination. Despite the distance of the modeled irradiance sensors from the system edges, the surrounding area still affects the results from a distance of more than 10 meters. This raises the question to what extent unit system simulations are representative of complete systems. Although we have shown that unit systems can be calibrated to account for the perimeter effect, these calibrations are only applicable at a specific location in the complete system.

We developed and applied a simulation parameters binning approach combined with a correction of the angle of incidence for direct sunlight. This approach alone reduced our simulation requirements by 88.2%, in addition to maintaining similar modeling accuracy. Further reduction in simulation requirements is possible with this approach, with only marginal impact on the modeling error.

With the calibrated unit system and binning approach, we achieved rRMSE values of 7.9% and 37.2% for the modeled front and rear irradiance, respectively. A front irradiance reference model achieved a similar performance of 8.0%. We therefore attribute at least part of this error to the systematic error introduced by the spatial separation of the direct and global irradiance input data and the on-site measurements of front and rear irradiance. Excluding approximately 10% of the data with suspected snow ground coverage reduced the rRMSE of the modeled rear irradiance to 18.9%.

The reduction of simulation requirements by binning depends on the location and system type. A typical dataset for a meteorological year, which is often used for modeling energy yields, consists of 8760 data points, including night hours. Energy yield simulations only require calculation of the daytime hours. For example, a typical meteorological year dataset in Wagga Wagga in Australia, has 4111 data points above global horizontal irradiance threshold of 30 W/m^2^. The same binning approach with a ‘Medium’ resolution and assuming a single-axis tracking system and constant albedo can reduce the number of simulations to 201, thus achieving an over 95% reduction in this case.

MCRT can provide valuable information for the accurate modeling of rear illumination in bifacial photovoltaic systems. Previous work already showed the impact of system components (Pelaez et al., 2019c; McIntosh et al., 2019), this work emphasizes the importance of accurate input assumptions such as the ground optical behavior.

Critically, we found that modeling the rear irradiance of this particular system requires time-resolved albedo inputs. Owing to periods of high albedo, the system cannot be accurately modeled using an average albedo parameter. If locally measured albedo data is not available, satellite-based data may be considered as a substitute. However, in our case, time-resolved satellite-data proved an ineffective replacement to ground measured data. This is likely because of the local character of the albedo, which is incompatible with the typically low spatial resolution of satellite-based albedo data.

Furthermore, the common assumption of isotropic ground reflectance may not be valid. At albedo values >30% for our reference data, the isotropic assumption leads to an overestimation of the modeled rear irradiance. MCRT is the ideal tool to reproduce anisotropic reflection properties. We have demonstrated that anisotropy can have a significant influence on the model results using the example of partial specular ground reflection.

The time-averaged ratio of rear irradiance to front irradiance received at the sensors is 11.0% for the full measured dataset. Therefore, for modules with a bifaciality factor of 80%, an energy yield gain of about 9% can be expected. Compared to measured data, our model yields a relative error of 6.4% for the time-averaged rear to front irradiance ratio. This error drops to 2.2% when periods of high albedo are excluded.

Finally, we demonstrated that accurate MCRT simulations of complex, full-scale PV installations can be addressed but require significant computational resources. In this work, we traced over 2.2 trillion rays (not counting the efforts in developing and tuning the MCRT simulations), which to the best of the authors' knowledge is the largest MCRT study published to date. This was possible through the support of HPC resources available to us through the Nectar Research Cloud and National Computational Infrastructure using up to 36,864 parallel compute cores.

Overall, this study provides a basis for simplifying bifacial illumination simulations which can lead to modeling the energy output of real bifacial tracking photovoltaic systems more accurately.

### Limitations of the study

This study validated simulation of front-illumination and rear illumination in a photovoltaic system using MCRT method. We demonstrated the impact of various modeling inputs, such as surrounding perimeter, ground albedo, and ground specularity on the simulation error. Impact of other extrinsic factors was not investigated, such as diffuse irradiance anisotropy, spatial non-uniformity of ground albedo, and surrounding structures. Similarly, simplifications were adopted regarding the spectral and angular behavior of the front and back of the solar modules because of the absence of relevant experimental data and to mitigate the model complexity.

## STAR★Methods

### Key resources table

**Table:** 

REAGENT or RESOURCE	SOURCE	IDENTIFIER
**Software and algorithms**		

Tracer	https://github.com/yosefm/tracer	
pvlib/pvlib-python: v0.8.1	https://doi.org/10.5281/zenodo.4417742	
Pre- and postprocessing scripts	https://github.com/e-marco	

### Resource availability

#### Lead contact

Further information and requests for resources and materials should be directed to and will be fulfilled by the lead contact, Marco Ernst (marco.ernst@anu.edu.au).

#### Materials availability

This study did not generate new unique materials.

### Method details

The modelling in this study was performed in a sequence of three parts. First, we prepared the input data in the pre-processing and data filtering step. Second, the MCRT modelling was performed using the open-source Python ray-tracer Tracer at its core. Finally, the generated simulation data was aggregated in a post-processing step. In the following, we describe these steps in detail.

The NREL field input dataset was downloaded from DuraMAT platform (Ayala Pelaez et al., 2020). At the time of access, the dataset included measurements from 01/06/2019 to 31/12/2020 with a total of 55,084 data rows.

Data pre-processing and filtering was applied to remove erroneous and/or unwanted entries:

1) Pre-processing: We dropped all unused columns from the dataset. For all remaining columns we then removed rows with invalid/empty or negative values. This reduced the number of rows to 15,807.
2) Removing unphysical values: We applied the BSRN rare limits, comparison filter and minimum global horizontal irradiance threshold of 30 W/m^2^ (Long and Dutton, 2021), using sun position and extra-terrestrial radiation computed with the pvlib library (Holmgren et al., 2021). These filters reduced the data to 14,530 rows.
3) Removing entries with suspected snow coverage:
  a. Greater than 30% difference between any of the available on-site albedo measurements
  b. Greater than 30% difference between any of the available on-site global horizontal irradiance measurements
  c. Greater than 30% difference between any of the available on-site front POA sensors

Due to the spatial difference of the global and direct irradiance measurements and on-site plane-of-array (POA) measurements, we also filtered data where the pvlib modelled POA deviates more than 30% from the measured on-site POA which affects 258 of the remaining rows, reducing the data to the final 5,326 rows.

From this dataset, we then generated the binned data for the resolutions listed in Table 1. The binning is applied in multiple dimensions separately for direct light (sun zenith, sun azimuth, tracking angle, ground albedo) and diffuse light (tracking angle, ground albedo). For the albedo measurements we used the IMT sensors (‘sunkitty_albedo_2’).

System simulations were then performed using Tracer. We designed the photovoltaic system using the existing capabilities of Tracer and implementing additional features, such as the Martin-Ruiz incidence angle modifier model and periodic boundary conditions. Each simulation consisted of several angular scenarios depending on the respective bin resolution and was further divided into smaller ray chunks. These sub-simulations were computed either sequentially or in parallel. The simulations resulted in the modelled irradiance at each of the five sensors at a nominal 1000 W/m^2^ irradiance for each unique bin.

MCRT belongs to the “embarrassingly parallel” computational problems category for which simulation wall time is typically reduced by parallel processing on a large number of CPUs. In this work, we utilised the high-performance computing (HPC) resources at the Australian National Computational Infrastructure (NCI) and Australian Research Data Commons (ARDC) Nectar Research Cloud. The code compatibility with commercial cloud-based HPC resources was also confirmed on a small dataset.

Data pre-processing and parameter binning was performed on a local computer using Python. The parallel MCRT simulation tasks were then processed on 96 to 36,864 parallel cores on the HPC resources using Python-based and MPI-based task queues in which each job simulated a small ray packet. The simulations required between 1 to 3 GB of RAM per core, depending on the complexity of the geometry and the size of the ray packets. To reduce storage requirements, we discarded unused results, such as ray direction information. Then, the individual simulation results were aggregated and synthesized into a human-readable format for analysis using Python.

We note that HPC is not essential to perform simulations with acceptable bin resolution. Benchmark simulations of the four-module unit system performed on an 8-core desktop computer (i7-70700 with 64 GB RAM) succeeded with over 390.000 rays per second (or 48.8 k-rays/sec per core). In comparison, the NCI infrastructure with Intel Xeon ‘Cascade Lake’ processors achieve a per-core performance of 84.3 k-rays/sec. A typical annual yield simulation at “Medium” bin resolution requires approximately 200 MCRT simulations. Thus, with 10 million rays for each of the MCRT simulations, the total simulation could be completed on the 8-core desktop computer in just over one hour. However, the complexity of the scene strongly affects the ray performance: Solving a full system with 200 modules and 20m perimeter reduces MCRT performance to approximately 5.5 k-rays/s per core. However, because in this case also far more rays per simulation are necessary, full system simulations are only feasible on HPC.

In the final step, these results were aggregated to expand the binned data back to the full input data of 5,326 rows. At each row of the input data, the respective simulation results for direct and diffuse irradiance were determined and scaled with the measured direct and diffuse irradiance, respectively.

The sensor values at each timestep t were calculated using the corresponding direct $E_{\text{sim},\text{direct}}$ and diffuse $E_{\text{sim},\text{diffuse}}$ simulation scaled by the measured direct normal irradiance DNI and diffuse horizontal irradiance DHI:

(Equation 4) $$E_{\text{sensor}}\left(t\right)=E_{\text{sim},\text{direct}}\times \frac{DNI\left(t\right)}{1000\text{W}/{\text{m}}^{2}}\times f+E_{\text{sim},\text{diffuse}}\times \frac{DHI\left(t\right)}{1000\text{W}/{\text{m}}^{2}}$$

The simulated sun position and tracking angles may differ from the annual dataset inputs, leading to a deviation between the simulated and actual angle of incidence at each timestep. To address this discrepancy, we applied an angle of incidence (AOI) correction factor $f=\frac{\cos\;AOI\left(t\right)}{\cos\;AOI_{\text{bin}}}$ to the direct irradiance simulation of the front sensors considering the angle of incidence of the sun on the tracker plane AOI relative to the angle of incidence $AOI_{\text{bin}}$ of the corresponding simulated bin. We assume f=1 for the rear sensors.

The post-processing results in a dataset which includes all the measured input data and additionally five modelled sensor irradiance values for each timestep.

### Quantification and statistical analysis

#### Stochastic uncertainty

In this work we compute the mean value $\bar{I_{N}}$, standard deviation $\sigma_{\text{N}}$, and the random simulation error $CI\left(I_{N}\right)$ with a 95% confidence level for a simulated irradiance by

(Equation 5) $$\bar{I_{N}}=\frac{1}{N}\sum_{i=1}^{N}I_{i}$$

(Equation 6) $$\sigma_{\text{N}}=\sqrt{\frac{1}{N}\sum_{i=1}^{N}\left(I_{i}-\bar{I_{N}}\right)}$$

(Equation 7) $$CI\left(I_{N}\right)=t^{*}\left(N-1\right)\frac{\sigma_{\text{N}}}{\sqrt{N-1}}$$

with $t^{*}\left(N-1\right)$ for two-tail t-distribution and 5% significance level ($t^{*}\left(\infty \right)=1.96$) Ref. (Beyer, 2017) and the number of simulations N>1. $I_{N}$ refers to a series of N independent simulations.

When computing the ratio of two MCRT simulation results – in our case the unit system and the reference system – both simulations are subject to statistical uncertainty. We thus calculate the resulting 95% confidence interval $CI\left(I_{p}^{*}\right)$ of $I_{p}^{*}$ by combination of the individual uncertainties using:

(Equation 8) $$CI\left(I_{p}^{*}\right)=\sqrt{{\left(\frac{CI\left(I_{N_{\text{max}},p}\right)}{\bar{I_{M_{\text{max}},\text{ref}}}}\right)}^{2}+{\left(-\frac{\bar{I_{N_{\text{max}},p}}}{{\bar{I_{M_{\text{max}},\text{ref}}}}^{2}}CI\left(I_{M_{\text{max}},\text{ref}}\right)\right)}^{2}}$$

#### Error metrics

Three error metrics are used to compare the simulated and measured irradiance of the five sensors (one facing the sun, four facing the ground), the relative Root Mean Square Error (rRMSE), relative Mean Bias Error (rMBE), and Coefficient of Determination ($R^{2}$):

(Equation 9) $$rRMSE=\frac{1}{\bar{O}}\sqrt{\frac{1}{n}\sum_{i=1}^{n}{\left(P_{i}-O_{i}\right)}^{2}}$$

(Equation 10) $$rMBE=\frac{1}{n\bar{O}}\sum_{i=1}^{n}\left(P_{i}-O_{i}\right)$$

(Equation 11) $$R^{2}=1-\frac{\sum_{i=1}^{n}{\left(O_{i}-P_{i}\right)}^{2}}{\sum_{i=1}^{n}{\left(O_{i}-\bar{O}\right)}^{2}}$$

$P_{i}$ represents the estimated value of the simulation of time *i*, $O_{i}$ represents the measured value at a given time *i*, *n* represents the total number of simulations, and $\bar{O}$ represents the mean of all the measurements. rRMSE measures accuracy as a percentage and is proportional to the size of the squared error; therefore, larger errors have a larger impact. rRMSE is converted to the units of the data by multiplying by the observed mean $\bar{O}$. rMBE provides the average error between the modelled and the measured value and is bias sensitive. $R^{2}$ similar to rRMSE is a measure of the squared error, but also assesses the quality of the model. For $R^{2}=1.0$, the simulation estimates and the observed values match perfectly while negative values indicate an absence of correlation between the model and the measured data.

## Data Availability

The MCRT ‘Tracer’ version used in this work is available in our GitHub repository https://github.com/e-marco. We are currently preparing our Python pre- and postprocessing scripts and filtered and binned data set used for the simulations for publication. The code will be available online via the same GitHub repository.
