# Supplementary material for: Accelerating the simulation of annual bifacial illumination of real photovoltaic systems with ray tracing
Source: iScience. 2021 Dec 25;25(1):103698. doi: 10.1016/j.isci.2021.103698 (PMC8760442; doi:10.1016/j.isci.2021.103698)
Supplement: Document S1. Figure S1 and Tables S1–S6 [file mmc1.pdf]

iScience, Volume *25*

Supplemental information

Accelerating the simulation of annual bifacial illumination of real photovoltaic systems with ray tracing

Marco Ernst, Georgia E.J. Conechado, and Charles-Alexis Asselineau

Supplementary Figures and Tables

Table S1. Geometric and optical parameters of the modelled bifacial system. Related to Figure 1.

Parameter	Value	Source
Ground-coverage ratio	0.35	(Pelaez et al., 2020)
Mounting height	1.5 m	(Pelaez et al., 2020)
Tracking angle	Calculated	Calculated using pvlib (PVLib, 2021; Holmgren et al., 2018) with 60° tracker limit and backtracking algorithm (Pelaez et al., 2020)
Site albedo	Measured<br>Isotropic (and partially specular) reflection	(Pelaez et al., 2020)
Module dimensions	0.99 m × 2.0 m	Assumed typical dimension for 72 cell / 144 half-cell modules
Torque tube	70% isotropic reflection<br>Circular, 0.12 m diameter	Own assumption
Post	70% isotropic reflection<br>Square, 0.09 m edge length	Own assumption
Module spacing	0.03 m	Own assumption
Module spacing over posts	0.25 m	Own assumption
Module front	Martin-Ruiz Incidence Angle Modifier with 10% specular reflection and a_r = 0.12	Own assumption
Module rear	70% isotropic reflection	Module row 3 is monofacial, adjacent rows bifacial (Pelaez et al., 2020)

Table S2. Time-resolved parameters utilised from the NREL dataset. Related to Figure 1.

Parameter	Unit	Data column
Direct normal irradiance	W/m^2	SRRL Direct CHP1-1 [W/m^2]
Diffuse horizontal irradiance	W/m^2	SRRL Diffuse 8-48 (vent) [W/m^2]
Site albedo	1	sunkitty_albedo_2
Front irradiance	W/m^2	poa_irradiance_front_IMT
Rear west irradiance	W/m^2	poa_irradiance_rear_IMT_West
Rear center west irradiance	W/m^2	poa_irradiance_rear_IMT_CenterWest
Rear center east irradiance	W/m^2	poa_irradiance_rear_IMT_CenterEast
Rear east irradiance	W/m^2	poa_irradiance_rear_IMT_East

Table S3. Error metrics of modelled front irradiance using time-resolved albedo at different bin resolutions. Related to Figure 7.

**Bin resolution**	**AOI correction**	***rRMSE*** **(%)**	***rMBE*** **(%)**	***rMAE*** **(%)**	R^2	$\bar{O}$ **(W/m²)**
Full	No	8.00	-2.31	5.27	0.96	657.1
Fine	No	8.15	-2.27	5.63	0.96	657.1
Medium	No	8.77	-2.62	6.41	0.95	657.1
Coarse	No	9.98	-3.23	7.62	0.93	657.1
Extra Coarse	No	10.70	-2.42	8.15	0.92	657.1
Full	Yes	7.99	-2.29	5.26	0.96	657.1
Fine	Yes	7.90	-2.18	5.22	0.96	657.1
Medium	Yes	7.87	-2.16	5.20	0.96	657.1
Coarse	Yes	7.81	-2.02	5.20	0.96	657.1
Extra Coarse	Yes	7.86	-1.99	5.18	0.96	657.1
PV Lib Reference	No	8.03	3.33	5.93	0.96	657.1

Table S4. Error metrics of modelled rear irradiance (average of four sensors) using time-resolved albedo at different bin resolutions. Related to Figure 8.

**Bin resolution**	**Albedo threshold**	***rRMSE*** **(%)**	***rMBE*** **(%)**	***rMAE*** **(%)**	R^2	$\bar{O}$ **(W/m²)**
Full	All	37.17	3.82	19.13	0.70	72.2
Fine	All	38.23	4.51	20.27	0.68	72.2
Medium	All	38.72	4.03	21.08	0.67	72.2
Coarse	All	41.41	4.87	21.61	0.63	72.2
Extra Coarse	All	39.17	4.49	20.99	0.67	72.2
Full	≤ 30%	18.90	-4.77	14.11	0.74	62.2
Fine	≤ 30%	20.51	-3.98	15.47	0.69	62.2
Medium	≤ 30%	21.91	-4.25	16.68	0.64	62.2
Coarse	≤ 30%	22.07	-3.99	16.61	0.64	62.2
Extra Coarse	≤ 30%	22.79	-2.87	16.94	0.61	62.2

Table S5. Error metrics of modelled rear irradiance (average of four sensors) using constant albedo at 'Medium' bin resolutions. Related to Figure 10.

Albedo parameter	Albedo threshold	*rRMSE* (%)	*rMBE* (%)	*rMAE* (%)	R^2	$\bar{O}$ (W/m^2)
**14%**	All	70.91	-34.64	36.69	-0.09	72.2
**16%**	All	67.26	-27.18	31.93	0.02	72.2
**18%**	All	64.53	-19.65	29.37	0.10	72.2
**20%**	All	62.82	-12.12	29.31	0.14	72.2
**22%**	All	62.27	-4.45	31.63	0.16	72.2
**24%**	All	62.96	3.22	35.51	0.14	72.2
**Satellite-albedo**	All	89.62	-42.44	45.30	-0.74	72.2
**Time-resolved albedo**	All	38.72	4.03	21.08	0.67	72.2
**14%**	≤ 30%	31.94	-23.21	25.83	0.24	62.2
**16%**	≤ 30%	26.27	-14.45	20.52	0.49	62.2
**18%**	≤ 30%	23.64	-5.61	18.03	0.59	62.2
**20%**	≤ 30%	25.04	3.23	18.75	0.54	62.2
**22%**	≤ 30%	30.11	12.24	22.51	0.33	62.2
**24%**	≤ 30%	37.41	21.23	28.25	-0.04	62.2
**Satellite-albedo**	≤ 30%	41.19	-27.08	30.51	-0.26	62.2
**Time-resolved albedo**	≤ 30%	21.91	-4.25	16.68	0.64	62.2

Table S6. Error metrics of modelled rear irradiance (average of four sensors) using time-resolved albedo at varying albedo specularity (applied to data with albedo > 30%). Related to Figure 11.

Specularity f_s (%)	*rRMSE* (%)	*rMBE* (%)	*rMAE* (%)	R^2	$\bar{O}$ (W/m^2)
**0**	38.72	4.03	21.08	0.67	72.2
**20**	36.41	2.70	20.06	0.71	72.2
**40**	37.01	1.42	19.96	0.70	72.2
**60**	40.24	0.17	20.72	0.65	72.2
**80**	45.44	-1.06	21.84	0.55	72.2
**100**	51.98	-2.26	23.28	0.41	72.2

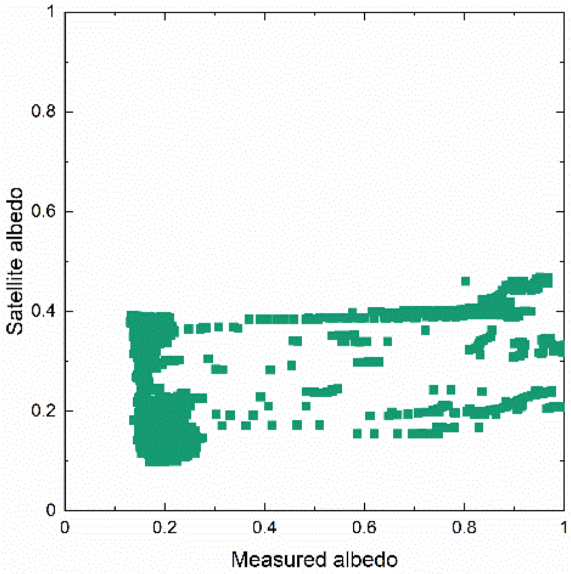

Figure S1. Satellite-albedo values versus ground-measured albedo values. Related to Figure 10.
